# COMMUNITY ENGAGEMENT IN THE DEVELOPMENT OF SEE ME: THE STUDENT-ELDER* ENGAGED MENTORING EXPERIENCE

**DOI:** 10.1093/geroni/igad104.3237

**Published:** 2023-12-21

**Authors:** Kimberly Oosterhouse, Lisa Skemp, Ginger Williams, Larry Lesperance, Jeanne Heid-Grubman, Caesar Hill, Hortense Banks-Wright

**Affiliations:** Loyola University Chicago, Chicago, Illinois, United States; Loyola University Chicago, Chicago, Illinois, United States; Edgewater Village Chicago, Chicago, Illinois, United States; Prime Timers, Chicago, Illinois, United States; Chicago Methodist Senior Services, Chicago, Illinois, United States; Hollywood House, Chicago, Illinois, United States; Hollywood House, Chicago, Illinois, United States

## Abstract

Loneliness, isolation, and suicide have been exacerbated by COVID-19 and ongoing uncertainties for both older adult and college-age populations. The 2023 Surgeon General’s Advisory on loneliness and isolation advocates for the healing effects of social connection, but ageist attitudes and stereotypes hinder social connections across generations. Intergenerational programs aim to address ageism and social connectivity, however, transferability of programs to culturally diverse groups remain challenging. Additionally, nursing curricula tend to focus on frail older adults, emphasizing vulnerability rather than wellness and disconnection rather than connection, contributing to disinterest in gerontological nursing. Prior community engaged ethnographic research identified five diverse older adult subcommunities that prioritized the need to address isolation and loneliness. Collaborating with these community organizations and nursing students, we developed the nursing Student-Elder*-Engaged Mentoring Experience (SEE ME) with the purpose of creating a sustainable and transferable program to address these needs. We will describe the research-based partnership process and resulting program development that focused on mutual wellness through reciprocal social connectivity among older adults in the community and nursing students in a required gerontological nursing course. In partnership, we developed and refined culturally appropriate processes, forms, assignments, activities, schedules, overall course organization, and program evaluation materials. Animated video modules and “Let’s Talk” questions were developed to facilitate student - older adult discussion. Dissemination and access to the program materials will be discussed. *The term “elder” was selected by the older adult community partners as their preferred nomenclature.

